# Reaching the malaria elimination goal in Brazil: a spatial analysis and time-series study

**DOI:** 10.1186/s40249-022-00945-5

**Published:** 2022-04-05

**Authors:** Gabriel Zorello Laporta, Maria Eugenia Grillet, Sheila Rodrigues Rodovalho, Eduardo Massad, Maria Anice Mureb Sallum

**Affiliations:** 1 Graduate Research and Innovation Program, Centro Universitario FMABC, Santo André, SP Brazil; 2 Laboratory of Parasite and Vector Biology, Institute of Zoology and Tropical Ecology, School of Sciences, Central University of Venezuela, Caracas, Venezuela; 3 Technical Unit of Transmissible Diseases and Current Health Assessment, Pan American Health Organization (PAHO/WHO), Brasília, DF Brazil; 4 School of Applied Mathematics, Getulio Vargas Foundation, Rio de Janeiro, RJ Brazil; 5 Epidemiology Department, School of Public Health, University of São Paulo, São Paulo, SP Brazil

**Keywords:** Malaria, Antimalarials therapeutic use, Brazil, Eradication, Prevention and control, Policy

## Abstract

**Background:**

Since 2015, the Global Technical Strategy (GTS) for Malaria 2016–2030 has been adopted by the World Health Organization (WHO) as a comprehensive framework to accelerate progress for malaria elimination in endemic countries. This strategy sets the target of reducing global malaria incidence and mortality rates by 90% in 2030. Here it is sought to evaluate Brazil’s achievements towards reaching the WHO GTS milestone in 2030. Considering the total number of new malaria cases in 2015, the main research question is: will Brazil reach the malaria elimination goal in 2030?

**Methods:**

Analytical strategies were undertaken using the SIVEP-malaria official databases of the Brazilian Malaria Control Programme for the Brazilian Amazon region from 2009 to 2020. Spatial and time-series analyses were applied for identifying municipalities that support the highest numbers of malaria cases over the years. Forecast analysis was used for predicting the estimated number of new cases in Brazil in 2025–2050.

**Results:**

Brazil has significantly reduced the number of new malaria cases in 2020 in comparison with 2015 in the states of Acre (− 56%), Amapá (− 75%), and Amazonas (− 21%); however, they increased in the states of Pará (156%), Rondônia (74%), and Roraima (362%). Forecast of the predicted number of new malaria cases in 2030 is 74,764 (95% *CI:* 41,116–141,160) in the Brazilian Amazon.

**Conclusions:**

It is likely that Brazil will reduce the number of new malaria cases in the Brazilian Amazon in 2030 in relation to that in 2015. Herein forecast shows a reduction by 46% (74,754 in 2030 forecast/137,982 in 2015), but this reduction is yet far from the proposed reduction under the WHO GTS 2030 milestone (90%). Stable and unbeatable transmission in the Juruá River Valley, Manaus, and Lábrea still support endemic malaria in the Brazilian Amazon. Today’s cross-border malaria is impacting the state of Roraima unprecedently. If this situation is maintained, the malaria elimination goal (zero cases) may not be reached before 2050. An enhanced political commitment is vital to ensure optimal public health intervention designs in the post-2030 milestones for malaria elimination.

**Graphical Abstract:**

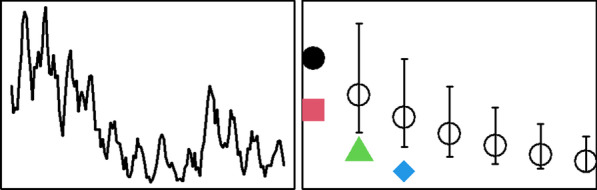

**Supplementary Information:**

The online version contains supplementary material available at 10.1186/s40249-022-00945-5.

## Background

The World Health Organization (WHO) Global Technical Strategy (GTS) for Malaria 2016–2030 is a comprehensive framework of strategies aligned to decrease malaria to defeat the disease in endemic countries. The strategies were developed with the ambitious target of reducing malaria incidence and mortality by at least 90% by 2030 [1]. The 2020 and 2025 WHO GTS milestones are reducing malaria mortality and incidence rates by 40% and 75%, respectively, in endemic countries [1]. Successful efforts have been implemented by Paraguay in 2018 followed by Algeria and Argentina in 2019 and El Salvador and China in 2021, which have been certified malaria-free [2–4]. In these countries the numbers of new malaria cases reached zero cases. In contrast, Nigeria, the Democratic Republic of the Congo, Uganda, Mozambique, and Niger account for approximately 51% of all cases globally, mainly those caused by *Plasmodium falciparum* [2]. This occurs because malaria transmission is active in these African countries and new malaria cases are consistently reported every year. In the WHO region of the Americas, Brazil, Colombia, Peru and Venezuela account for 90% of the share of malaria cases, with Colombia and Peru recently committing to accelerate progress towards malaria elimination in South America [5, 6]. However, the epidemiological situations in Brazil and Venezuela are worrying. The figures of new malaria cases increased by 3% from 2015 (138,004) to 2020 (142,124) [7] in Brazil, which represents a frustrating outcome in regard to the proposed WHO 2020 milestone of reducing malaria incidence by at least 40% compared with that in 2015 [1]. In addition, Venezuela has experienced a socioeconomic, political and health care crisis in the last decade that has promoted substantial migration of Venezuelans to neighbouring countries and catalysed the overflow of infectious diseases, such as malaria [8]. To further illustrate the complexities related to eliminating the disease, Venezuela was certified by the WHO as malaria-free 60 years ago in June 1961 [9], but the disease upsurged in the last decade (2011–2020) due to erratic political decisions, negative economic growth rates, and shortcomings in people’s access to health commodities [8, 10].

In accordance with the WHO GTS for Malaria and the Sustainable Development Goals (SDGs), the Brazilian Ministry of Health (BMoH) launched the malaria elimination plan, with emphasis on *P. falciparum* [11]. The total number of new *P. falciparum*-malaria cases increased by 49% from 2015 (15,433) to 2020 (23,033) in Brazil [7]. The majority of new malaria cases in Brazil are associated with *P. vivax* [12–14]. In 2015, Brazil reported a total of 138 004 new malaria cases, with 121,217 (87.8%) of *P. vivax*, 15,433 (11.2%) of *P. falciparum*, and 1354 (1%) of *Plasmodium malariae* or unidentified [7]. The number of cases in Brazil was 142,124 in 2020 sorted by 118,959 *P. vivax* (83.7%), 23,033 *P. falciparum* (16.2%), and 132 *P. malariae* or unidentified (0.1%) [7]. Additionally, malaria transmission occurs outside the Brazilian Amazon Region [15–17]. Only 90 (0.07%) and 19 (0.01%) malaria cases occurred in the extra-Amazon region in 2015 and 2020, respectively [18]. Despite the very low figures, the extra-Amazonian region importance relies on its vulnerability to the reintroduction of cases, particularly *P. vivax* malaria [16].

Spatial statistics and time series analyses are key tools for modelling the impact of interventions adopted for malaria elimination [19]. The identification of clusters of epidemic municipalities can be used as a decision support tool for the control of malaria transmission in the Brazilian Amazon [20]. Spatially associated malaria case incidence indicates geographical priority for reducing the suffering from local people and the risk of malaria spillover to bordering areas [21, 22]. Time series analysis can be used to forecast predicted number of new malaria cases in the states of Brazilian Amazon [23]. Autoregressive integrated moving average (ARIMA) forecasting tool can be applied for the prediction of future time series of malaria cases to assist in the delineation of control strategies [23].

Considering the drop from approximately 600 thousand new malaria cases in 2006 to ~ 150 thousand in 2019, the main research question here is: will a malaria-free level ever be reached in Brazil? Taking the WHO GTS 2030 milestone into consideration, a 90% malaria incidence case reduction compared with that in 2015 indicates a maximum of 13 800 new malaria cases in Brazil by 2030 [1]. To help to understand the feasibility of Brazil achieving the malaria elimination goal by 2030, this study aims to: (1) identify clusters of epidemic municipalities in the Brazilian Amazon during 2009–2020, (2) assess temporal stability of the number of new malaria cases in these municipalities, (3) analyse the impact of cross-border malaria, and (4) forecast the predicted number of new malaria cases in 2025 and 2030.

## Methods

### Epidemiological data

The SIVEP-malaria notification databases from 2009 to 2020 were obtained from the Brazilian Malaria Control Programme through the Law of Access to Information, protocol no. 25072.022472/2020-52.

Specific datasets were retrieved from the SIVEP-malaria databases using the *R* programming language v. 4.0.4 package *tidyverse* v. 1.3.1 (tidyverse.org/packages/) [24]. Missing data were excluded from analyses because they were negligible and represented less than 0.01% of the variables filtered in the datasets as follows: (1) Dataset to identify clusters of epidemic municipalities. All positive malaria tested by light microscopy were filtered according to:

Filter 1, *country of infection* = Brazil, it means malaria infection occurred in Brazil. Filter 2, *state of infection* = Acre, Amapá, Amazonas, Maranhão, Mato Grosso, Pará, Rondônia, Roraima, and Tocantins; it means malaria infection occurred in the Brazilian Amazon region. Filter 3, *new case only* = YES, it means other cases were excluded (cure check testing). Filtered malaria cases were grouped and summarized by municipalities of infection per year (Additional file 1). (2) Dataset to assess temporal stability of epidemic municipalities. All positive malaria tested by light microscopy were filtered according to:

Filter 1–Filter 3, as above mentioned. Filter 4, *municipality of infection* = Bagre, Barcelos, Coari, Cruzeiro do Sul, Guajará, Ipixuna, Lábrea, Manaus, Mâncio Lima, Oeiras do Pará, Rio Preto da Eva, Rodrigues Alves, and Santa Isabel do Rio Negro, as these municipalities were identified as epidemic clusters in the previous analysis. Filtered malaria cases were grouped and summarized by month and year of official case report per municipality of infection (Additional file 2). (3) Datasets to analyse the impact of cross-border malaria. Imported malaria cases per country of infection were filtered by: Filter 5, *country of infection* = Bolivia, Colombia, Guyana, French Guiana, Peru, Suriname, and Venezuela, which means malaria infection occurred in bordering countries to the Brazilian Amazon. Filter 6, *state of official case report* = Acre, Amapá, Amazonas, Maranhão, Mato Grosso, Pará, Rondônia, Roraima, and Tocantins; these Brazilian Amazon states received the burden of having to diagnose and treat imported malaria cases free-of-charge. Filter 7, *new case only* = YES, it means other cases were excluded (cure check testing). Filtered malaria cases were grouped and summarized by country of infection per year (Additional file 3). Imported malaria cases per municipality of official case report were filtered by Filter 5–Filter 7, then grouped, and summarized by municipality of official case report per year (Additional file 4). Imported *P. falciparum* malaria cases per municipality of official case report were filtered by Filter 5–Filter 7 and by Filter 8, *parasite species diagnosis* = *P. falciparum* only, *P. falciparum* + *P. vivax*, or *P. falciparum* + *P. malariae*. Filtered *P. falciparum* malaria cases were grouped and summarized by municipalities of official case report per year (Additional file 5). (4) Dataset to forecast the predicted number of new malaria cases. All new malaria cases in all Brazilian Amazon states were grouped and summarized by month and year of official case report (Additional file 6). (5) Dataset for exploratory description. All *P. vivax* malaria cases were filtered by *country of infection* = Brazil; *state of infection* = Acre, Amapá, Amazonas, Maranhão, Mato Grosso, Pará, Rondônia, Roraima, and Tocantins; *new case only* = YES; and *parasite species diagnosis* = *P. vivax* only. All *P. falciparum* malaria cases were filtered by these same filters, except *parasite species diagnosis* = *P. falciparum* only, *P. falciparum* + *P. vivax*, or *P. falciparum* + *P. malariae*. They were grouped and summarized per year (Additional file 7).

### Identification of clusters of epidemic municipalities

The total number of new malaria cases reported by municipality of infection were calculated for all Brazilian Amazonian municipalities (*n* = 808) from 2009 to 2020. The division of the Brazilian territory per municipality administrative border was projected into topographic mapping with the SIRGAS 2000 datum and Brazil polyconic projection (EPSG 5880). The spatial weight matrix (SWM) for the topographic mapping was calculated. An inverse distance weighting with a negative exponential function of distance of the formula was used:

$${w}_{ij}=\mathrm{exp}(-\alpha {d}_{ij})$$

where *w* = spatial weight matrix, *d*_ij_ = centroid distances between each pair (*ij*) of country municipalities and *α* = parameter of spatial weight. The selected value of *α* = 5 was considered for the calculation of the spatial weight matrix. This value of *α* = 5 means that the spatial weight from a focal municipality (*i*) to its neighbouring municipalities (*j*) decayed exponentially with distance. This means that the event of interest (the number of malaria cases per municipality of infection) has a considerably high spatial autocorrelation, as supported by a previous study showing the high levels of spatial aggregation of malaria transmission in the Brazilian Amazon [21]. The centroid distances were calculated with the Euclidean (straight-line) distance method. The threshold distance value for the calculation of spatial weights was specified as 250 km.

The local indicator of spatial association (LISA) was used to detect clusters of high values of new malaria cases. The LISA method was applied to calculate the local Moran statistic (*I*_i_) as follows:

$${I}_{i}=\frac{{z}_{i}\sum_{j}{w}_{ij}{z}_{j}}{c}$$

which corresponds to the product of the value of new malaria cases at location *i* with its spatial lag—the weighted sum of the values at neighbouring locations, where *z*_i_ and *z*_j_ = deviations of values from the mean new malaria cases at the focal municipality and its neighbours, *w*_ij_ = spatial weight matrix and *c* = the variance of new malaria cases for standardization purposes. Hypothesis testing of the local Moran statistic *I*_i_ was performed to calculate the statistical significance based on a false discovery rate correction for a 95 percent confidence interval (95% *CI*). Four categories of clusters were mapped. Clusters with high values of new malaria cases in the focal municipality and neighbours were classified as high–high clusters; otherwise, they were classified as low–low clusters. Outliers were identified if the focal municipality had a high value of new malaria cases and its neighbours had low values (high-low outlier), or vice versa. Because the spatial distribution of malaria in Brazil is highly unequal [21], the top 1.9% of the 808 municipalities (*n* = 15) per year were identified based on the high values of the local Moran statistic (*I*_i_). Those that were identified as ≥ 50% over the years were considered epidemic municipalities of malaria transmission in Brazil.

### Assessment of temporal stability of epidemic municipalities

Monthly new malaria cases from January 2009 to December 2020 (*Y*_1−144_) reported in the selected municipalities were firstly correlated to each other using Spearman’s rank correlation test (*α* = 0.05). The time series (*Y*_1−144_) per municipality was then adjusted to a seasonal autoregressive integrated moving average model (seasonal ARIMA). The seasonal ARIMA was calculated using the following formula:

$${Y}_{t}= c+{\phi }_{p} {Y}_{t-1}+{(1-{Y}_{t-1})}^{d}+{{\theta }_{q}\epsilon }_{t-1}+{\Phi }_{P}{Y}_{t-1}^{m}+{(1-{Y}_{t-1}^{m})}^{D}+{{\Theta }_{Q}\epsilon }_{t-1}^{m}$$

where the number of monthly malaria cases (*Y*_t_) is a function of a constant drift (*c*) plus a linear combination lag of malaria cases of order *p* (${\phi }_{p} {Y}_{t-1}$) plus a linear combination of lagged error of order *q* (${{\theta }_{q}\epsilon }_{t-1}$) plus seasonal (${\Phi }_{P}{\Theta }_{Q}$) and integrative (*d*, *D*) parameters for a given seasonal frequency *m*. Further information on the application of seasonal ARIMA on monthly malaria cases in Brazil can be found elsewhere [23].

Hypothesis testing was carried out to determine if the monthly trend (${\phi }_{p} {Y}_{t-1}$) in malaria cases in the time series was an increase (emergence), a stabilization (control) or a decrease (reduction). If ${\phi }_{p}$ = 0, then the malaria trend was stabilized (control), whereas ${\phi }_{p}$ > 0 was an increase (emergence) and ${\phi }_{p}$ < 0 was a decrease (reduction), considering a level of confidence of 5%. Estimated parameters of seasonal ARIMA were used to predict monthly malaria cases in the forecasting horizon of 12 months to provide further insights into the malaria trends in 2021.

### Analysis of the impact from cross-border malaria

New malaria cases that were infections from Bolivia, Colombia, Guyana, French Guiana, Peru, Suriname, and Venezuela, but were diagnosed and treated in Brazil, were analysed into three approaches: (1) calculation of the ratio between the proportion of imported vs the proportion of autochthonous *P. falciparum*-malaria cases to estimate the impact of imported falciparum malaria cases; (2) mapping of imported malaria cases per municipality of official case report to estimate their impact on the local malaria public health facilities; and (3) statistical testing of the difference of the number of imported malaria cases per country using Welch’s two-sample *t*-test (*α* = 0.05).

### Forecast of the predicted number of new malaria cases

Monthly new malaria cases (*Y*_1−144_) in the Brazilian Amazon were adjusted into a seasonal ARIMA model [23]. The adjusted seasonal ARIMA model was used to forecast the total number of malaria cases in the Brazilian Amazon in 2025 and 2030. The predicted values in 2025 and 2030 were compared with the expected values, respectively, as follows: (1) the total number of new malaria cases in 2015 multiplied by 25% (a 75% reduction under the WHO GTS milestone in 2025), and (2) the total number of new malaria cases in 2015 multiplied by 10% (a 90% reduction under the WHO GTS milestone in 2030) [1].

## Results

### Descriptive analysis

**Fig. 1:**
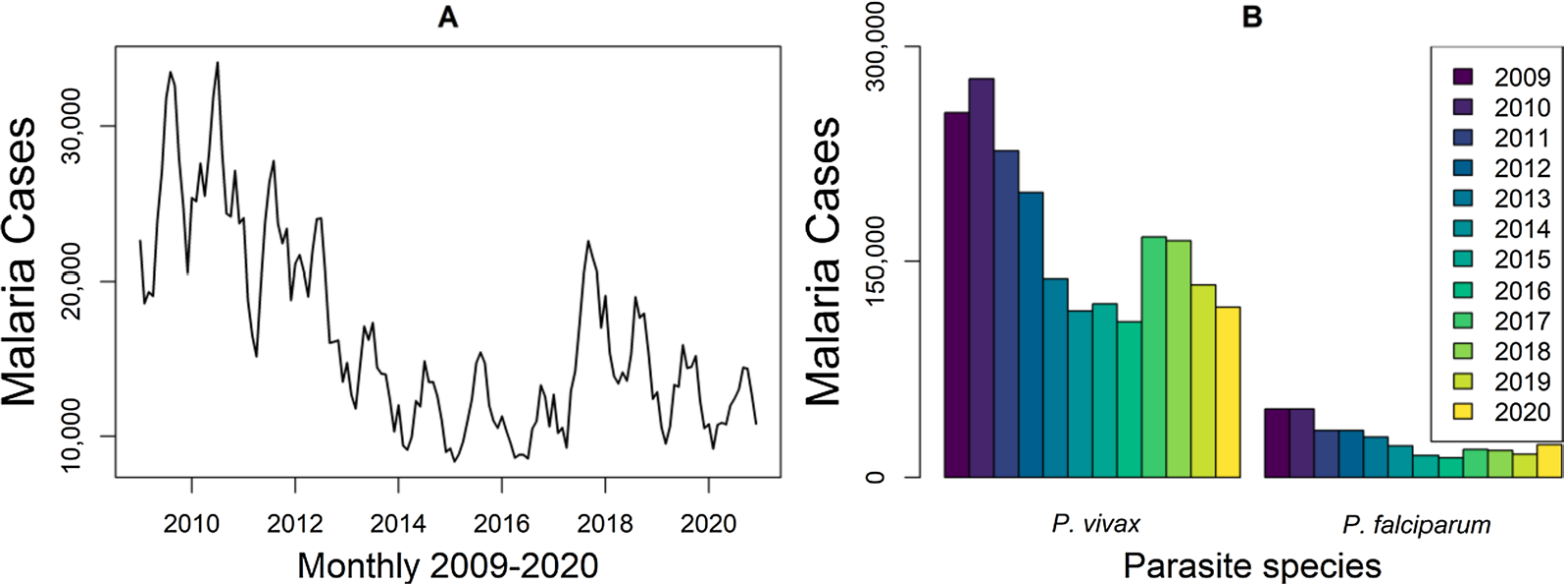
Number of new malaria cases in the nine states of the Brazilian Amazon. **A** Monthly new malaria cases 2009-2020. **B** Annually new malaria cases per parasite species

In the 9 states and 808 municipalities of the Brazilian Amazon, the total number of new malaria cases decreased by 56% from 301,581 in 2009 to 137,982 in 2015 and increased by 15% from 123 905 in 2016 to 142,112 in 2020 (Fig. 1A). The proportions of *P. vivax* vs *P. falciparum* new malaria cases varied 83–90% vs 10–17% 2009–2020 (Fig. 1B).

**Fig. 2:**
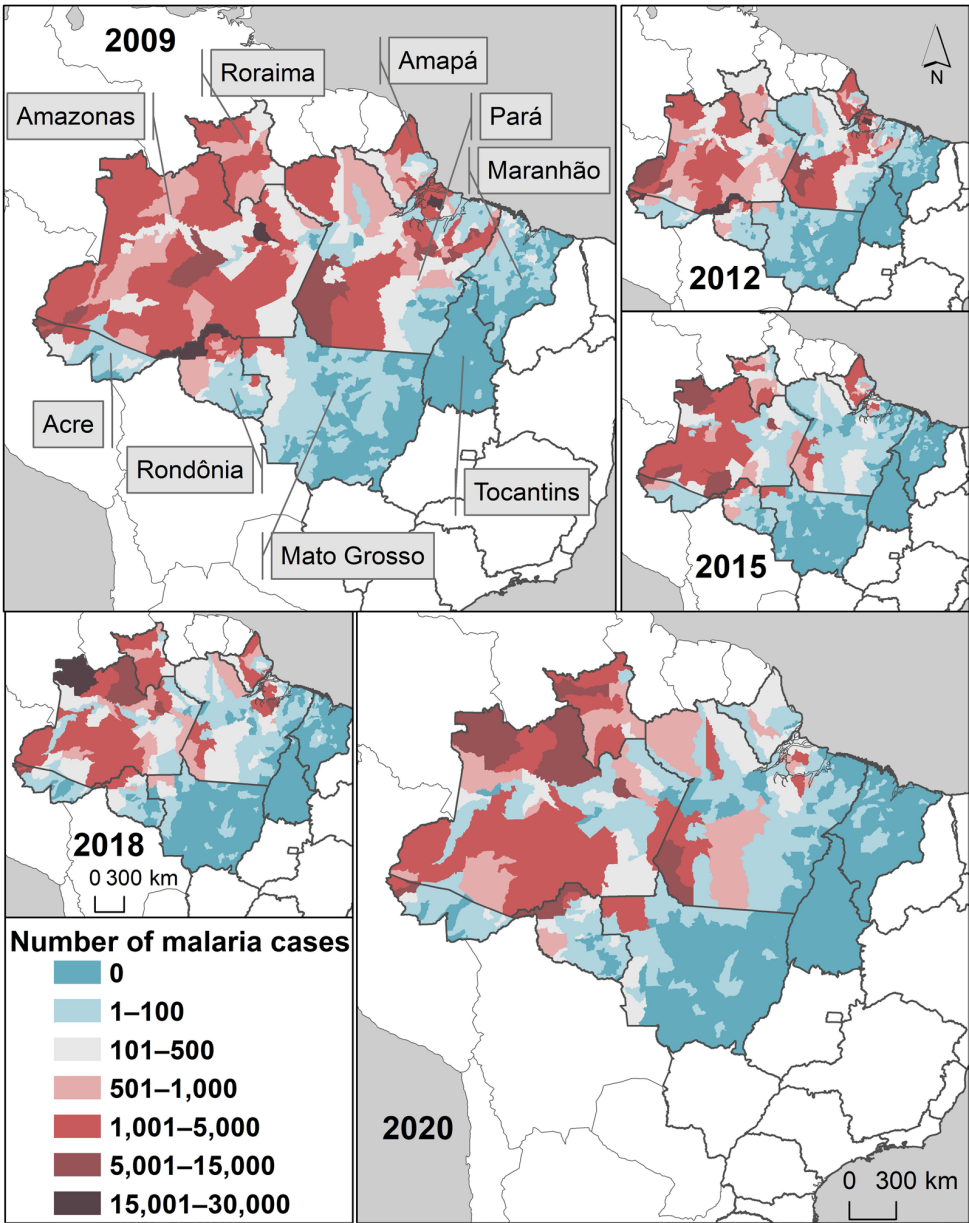
Number of new malaria cases in the nine states of the Brazilian Amazon

The nine Brazilian states in the Amazon Region—Acre, Amapá, Amazonas, Maranhão, Mato Grosso, Pará, Rondônia, Roraima, and Tocantins had variations in the number of new malaria cases per year (Fig. 2). Variations in 2009–2015 and in 2015–2020 were + 3% and − 56% in Acre, + 3% and − 75% in Amapá, − 27% and − 21% in Amazonas, − 96% and − 60% in Maranhão, − 63% and + 202% in Mato Grosso, − 90% and + 156% in Pará, − 83% and + 74% in Rondônia, − 54% and 362% in Roraima, and − 100% in Tocantins (Fig. 2).

### Specific aim 1: clusters of epidemic municipalities

**Fig. 3:**
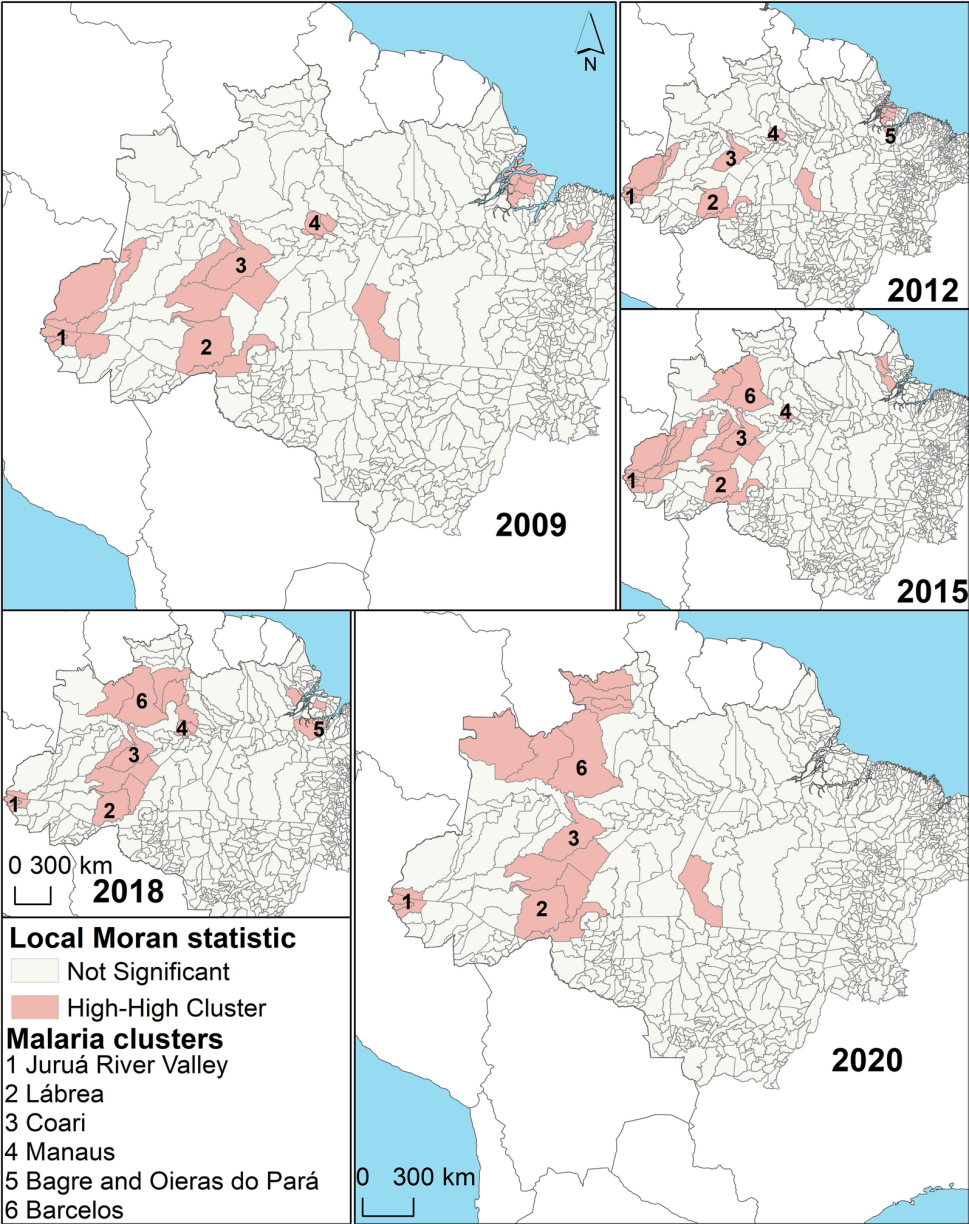
Local Moran statistic (*I*_i_) high–high clusters of new malaria cases in the selected years for Brazil

Based on the new malaria cases per municipality in Fig. 2, cluster analysis was carried out per year to obtain the number of high-high clusters (focal and neighbouring municipalities with high numbers of malaria cases) per year. Figure 3 shows clusters of epidemic municipalities (high-high clusters) in 2009, 2012, 2015, 2018, and 2020, labelled with numbers: **1**, Juruá River Valley (Cruzeiro do Sul, Mâncio Lima, Rodrigues Alves e Guajará); **2**, Lábrea; **3**, Coari; **4**, Manaus; **5**, Bagre and Oieras do Pará; and **6**, Barcelos. The others, unlabelled high–high clusters are transient hotspots (see definition in Table 1).

**Table 1:**
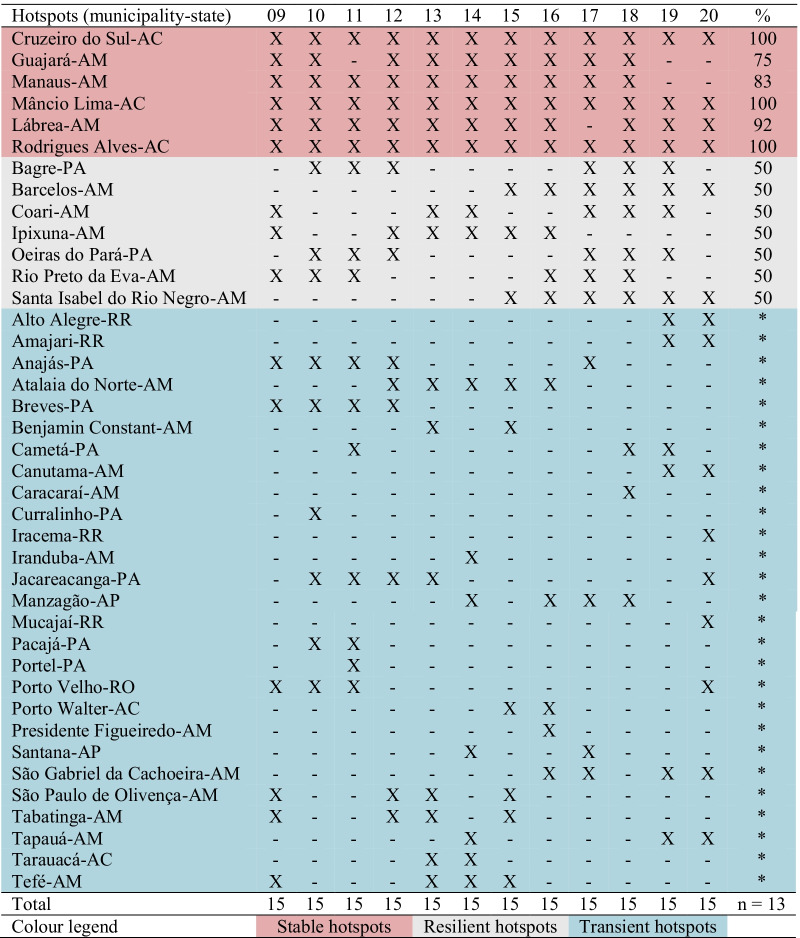
Results from the local Moran statistic to detect epidemic municipalities in the Brazilian Amazon from 2009 to 2020

X: municipalities having the highest values of local Moran statistic (*I*_i_); -: the lowest values of *I*_i_

50–100%: identification of epidemic municipalities (*n* = 13); *: otherwise, < 50% over the years

*AC* Acre state; *AM* Amazonas state; *RR* Roraima state; *AP* Amapá state; *PA* Pará state; *RO* Rondônia state

In each year, 15 municipalities with the highest values of the local Moran statistic were selected from the pool of high–high clusters and were denominated as the epidemic municipalities of the year (Table 1). The selected municipalities varied substantially per year; however, several municipalities were frequently selected. Cruzeiro do Sul, Mâncio Lima, and Rodrigues Alves in Acre state were chosen in all years, and Manaus, Lábrea, and Guajará in Amazonas state were in > 80% of the period. Bagre-PA, Barcelos-AM, Coari-AM, Ipixuna-AM, Oieras do Pará-PA, Rio Preto da Eva-AM, and Santa Isabel do Rio Negro-AM were selected in 50% of the years analysed. Together, these municipalities were demonstrated to be hotspots of malaria transmission (Table 1). Stable hotspots (red) are municipalities that maintain the annual number of malaria cases within a fixed range over the years. Resilient hotspots (grey) are municipalities that have a short period of fewer cases in a stable time series of malaria cases. Transient hotspots (blue) are municipalities that show one or more peaks of an increased number of cases but often have no or low malaria case incidence (Table 1).

### Specific aim 2: temporal stability of epidemic municipalities

**Fig. 4:**
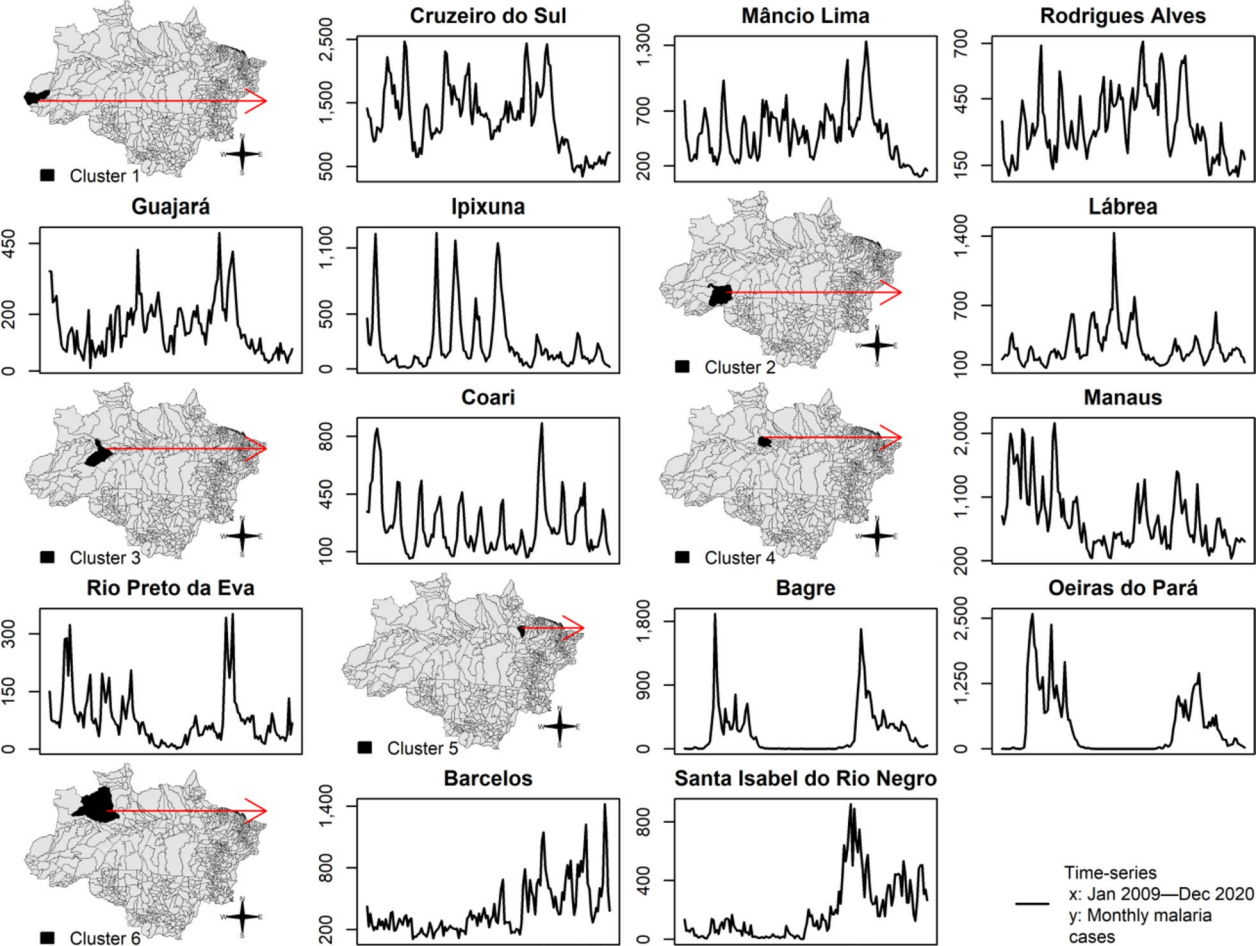
Time series of monthly new malaria cases in epidemic municipalities in Brazil, 2009–2020

Figure 4 shows time-series of monthly new malaria cases in the epidemic municipalities. The red arrows link geographical locations with time series of each epidemic municipality in Brazil as follows: Cluster 1 = Cruzeiro do Sul, Mâncio Lima, Rodrigues Alves, Guajará, and Ipixuna; Cluster 2 = Lábrea; Cluster 3 = Coari; Cluster 4 = Manaus and Rio Preto da Eva; Cluster 5 = Bagre and Oeiras do Pará; and Cluster 6 = Barcelos and Santa Isabel do Rio Negro (Fig. 4). Monthly new malaria cases were autocorrelated per municipality in each cluster, as follows: Cruzeiro do Sul and Mâncio Lima (*rho* = 0.77, *P* < 0.001), Rodrigues Alves (*rho* = 0.69, *P* < 0.001), and Guajará (*rho* = 0.58, *P* < 0.001) in Cluster 1; Manaus and Rio Preto da Eva in Cluster 4 (*rho* = 0.59, *P* < 0.001), Bagre and Oeiras do Pará in Cluster 5 (*rho* = 0.93, *P* < 0.001), and Barcelos and Santa Isabel do Rio Negro in Cluster 6 (*rho* = 0.66, *P* < 0.001).

Time-series of monthly new malaria cases of each epidemic municipality in Fig. 4 was adjusted into a seasonal ARIMA model. Table 2 shows results of the autoregressive parameter of the adjusted seasonal ARIMA model per epidemic municipality. All the six clusters had epidemic municipalities with an increasing trend in monthly new malaria cases (Table 2). A total of 9 out of 13 (69%) epidemic municipalities (Bagre, Barcelos, Coari, Guajará, Ipixuna, Lábrea, Manaus, Santa Isabel do Rio Negro, and Rodrigues Alves) showed an increasing trend in monthly new malaria cases (Table 2). Three municipalities, (Cruzeiro do Sul, Mâncio Lima, and Rio Preto da Eva) showed a decreasing trend in monthly new malaria cases, while Oeiras do Pará showed a stable trend (Table 2).

**Table 2:** Results of seasonal ARIMA model in epidemic municipalities, Brazil, 2009–2020

Municipality	Parameter (ar)^a^	Estimate	*P*	Interpretation
*Cluster 1*				
Cruzeiro do Sul	${\phi }_{1}$	0.04	0.64	Decreasing
	${\phi }_{2}$	0.05	0.54	
	${\phi }_{3}$	− 0.27	< 0.001	
Mâncio Lima	${\phi }_{1}$	1.71	< 0.001	Decreasing
	${\phi }_{2}$	− 0.92	< 0.001	
Rodrigues Alves	${\phi }_{1}$	0.67	< 0.001	Increasing
Guajará	${\phi }_{1}$	0.72	< 0.001	Increasing
Ipixuna	${\phi }_{1}$	0.62	< 0.001	Increasing
*Cluster 2*				
Lábrea	${\phi }_{1}$	0.78	< 0.001	Increasing
*Cluster 3*				
Coari	${\phi }_{1}$	0.89	< 0.001	Increasing
*Cluster 4*				
Manaus	${\phi }_{1}$	0.68	< 0.001	Increasing
Rio Preto da Eva	${\phi }_{1}$	0.50	< 0.05	Decreasing
	${\phi }_{2}$	− 0.77	< 0.001	
*Cluster 5*				
Bagre	${\phi }_{1}$	0.84	< 0.001	Increasing
Oeiras do Pará	${\phi }_{1}$	Absent	Absent	Stable
*Cluster 6*				
Barcelos	${\phi }_{1}$	0.59	< 0.001	Increasing
Santa Isabel do Rio Negro	${\phi }_{1}$	0.45	< 0.05	Increasing

^a^*ar* autoregressive parameter in seasonal ARIMA

**Fig. 5:**
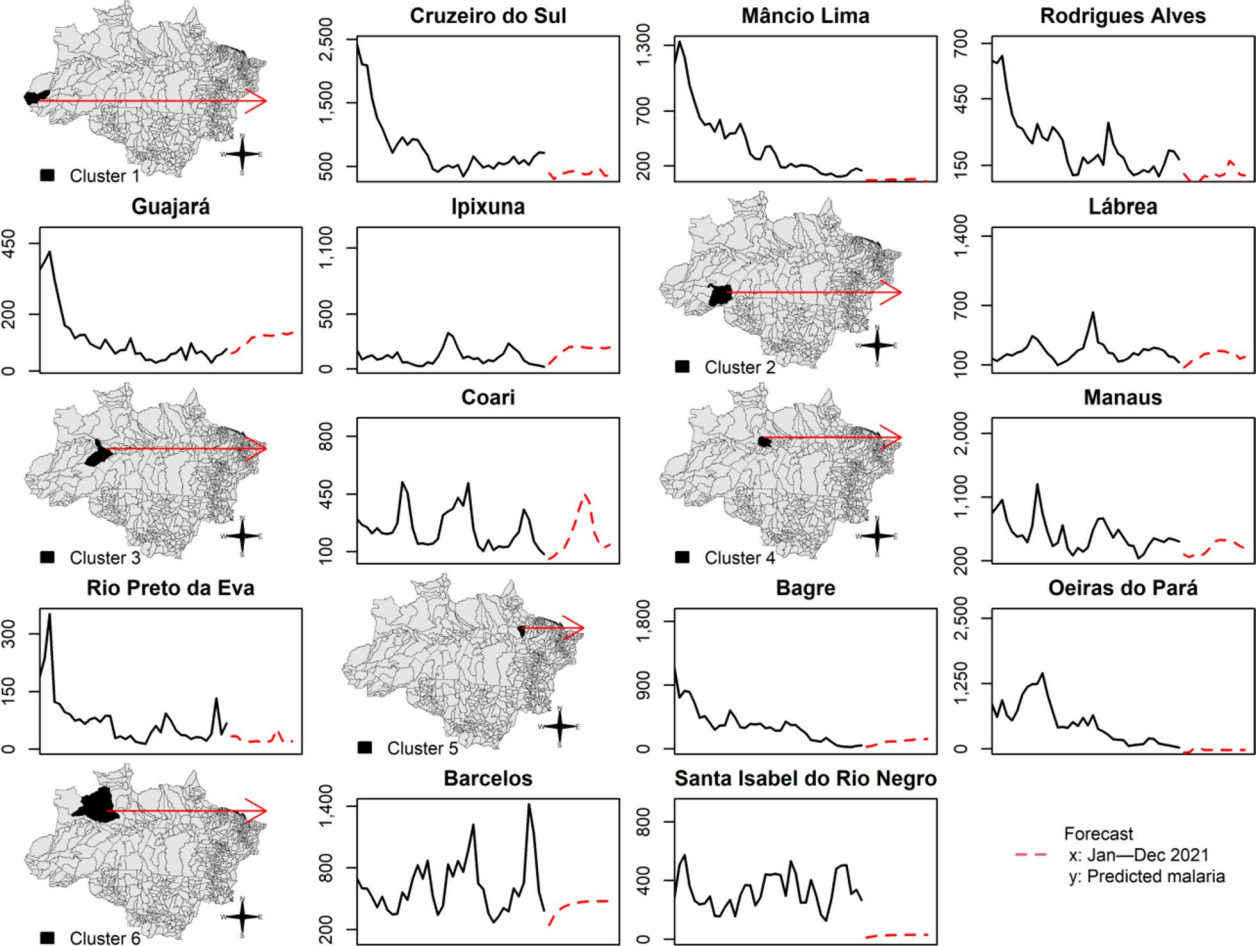
Forecast of monthly new malaria cases from January to December 2021

The adjusted ARIMA model was used to forecast the predicted number of monthly new malaria cases in 2021 per epidemic municipality (Fig. 5). The dashed lines in Fig. 5 represent the predicted number of monthly new malaria cases in 2021. Coari, Guajará, and Ipixuna will likely present higher levels of monthly new malaria cases in 2021 (Fig. 5).

### Specific aim 3: impact from cross-border malaria

**Fig. 6:**
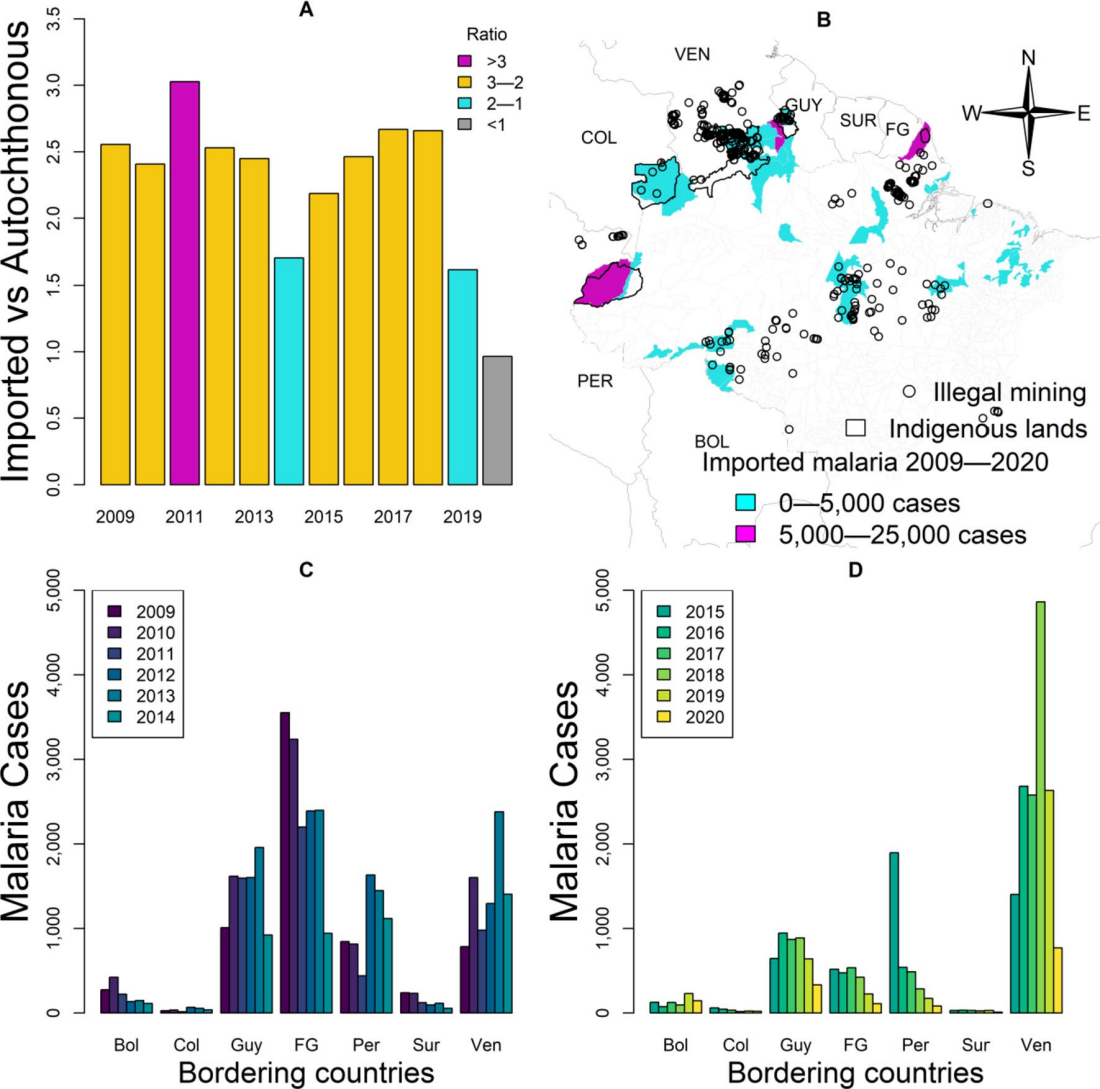
Analysis of imported malaria cases. **A** Ratio between the proportions of imported vs. autochthonous falciparum malaria cases per year. **B** Brazilian municipalities that reported a total of 59,480 imported malaria cases from 2009 to 2020. **C** Distribution of the total number of imported malaria cases per country of infection Bolivia (Bol), Peru (Per), Colombia (Col), Venezuela (Ven), Guyana (Guy), Suriname (Sur), and French Guiana (FG). **D** Distribution of the total number of imported malaria cases per country of infection in 2015–2020

The proportions of imported vs autochthonous *P. falciparum* malaria cases were 41% (2727/6729) vs 16% (47,841/301,581) in 2009, 25% (1143/4674) vs 11% (15,432/137,982) in 2015, and 14% (206/1470) vs 15% (18,017/124,185) in 2020. The mean proportion of imported *P. falciparum* malaria cases was 29.6% (95% *CI:* 12.6–46.6), while the mean proportion of autochthonous *P. falciparum* malaria cases was 13.2% (95% *CI:* 8.4–17.9). Figure 6A shows the ratio between the proportions of imported vs autochthonous *P. falciparum* malaria for all years. This ratio was 2.6 (41%/16%) in 2009, 2.3 (25%/11%) in 2015, and 0.9 (14%/15%) in 2020. It was above 1 in all years, except in 2020 (Fig. 6A). The ratio above 1 indicates a greater proportion of imported than autochthonous *P. falciparum* cases.

A total of 59,480 imported and of 2,363,779 autochthonous malaria cases were reported from 2009 to 2020 in the Brazilian Amazon. The imported cases were diagnosed and treated in a total of 51 municipalities during this period (Fig. 6B). At least 16 (31.3%) of these municipalities are country-border municipalities (Fig. 6B), such as Guajará-Mirim and Porto Velho in Rondônia state (Bolivian border); Acrelândia and Plácido de Castro in Acre state (Bolivian border); Atalaia do Norte, Benjamin Constant, Tabatinga and São Gabriel da Cachoeira in Amazonas state (Peruvian, Colombian, and Venezuelan borders); Alto Alegre, Iracema, Caracaraí, Bonfim, Uiramutã, Paracaima and Amajari in Roraima state (Venezuelan and Guyana borders); and Oiapoque in Amapá state (French Guiana border). In addition, indigenous lands on the Brazilian border and illegal mining locations overlap with these municipalities (Fig. 6B).

Figure 6C shows that higher numbers of imported malaria cases were from French Guiana (14,717), Guyana (8706), Venezuela (8447), and Peru (6295) from 2009 to 2014. The pairwise hypothesis testing showed that the comparison between French Guiana and every other country was statistically significant (*t* > 2.3, *P* < 0.05) from 2009 to 2014 (Fig. 6C). From 2015 to 2020, imported malaria cases from Venezuela increased 77% (14,925) in comparison with the previous period, while imported cases from French Guiana (2286), Guyana (4321), and Peru (3431) decreased (Fig. 6D). The number of imported malaria cases from Venezuela was statistically higher than those from every other country (*t* > 3, *P* < 0.05) from 2015 to 2020. Furthermore, the average number of imported cases from 2009 to 2019 was 5932 (95% *CI* 2909–8955), while in 2020, it decreased to 1470, which may be related to the COVID-19 pandemic year, because of cross-border mobility restrictions (Fig. 6D).

**Fig. 7:**
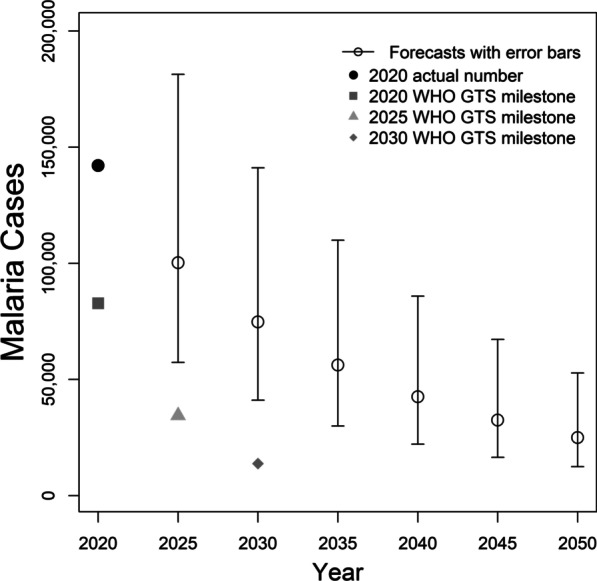
Forecast of the predicted number of new malaria cases in 2025 and 2030 in comparison with the WHO GTS milestones for 2025 and 2030

### Specific aim 4: predicted number of new malaria cases

The total number of new malaria cases in 2015 in the Brazilian Amazon was 137,982. According to the WHO GTS milestones, the targeted numbers of new malaria cases are 82,789 in 2020 (40% reduction), 34,496 in 2025 (75% reduction), and 13,798 in 2030 (90% reduction in relation to that in 2015). The number of malaria cases in 2020 was 142,107 which is 72% above the target (82,789) under the 2020 WHO GTS milestone (Fig. 7). The predicted values of new malaria cases were 100 306 (95% *CI:* 57,319–181,386) in 2025 and 74,765 (95% *CI*: 41 116–141,160) in 2030, which are 190% and 441% above the targets 34,496 and 13,798 for the 2025 and 2030 WHO GTS milestones, respectively (Fig. 7). The 2020 WHO GTS milestone can be achieved until 2030, while the 2030 WHO GTS milestone will not be achieved before 2050 (Fig. 7).

## Discussion

In the last two decades, malaria control programmes carried out in Brazil have shown the capacity to establish long-term targets for achieving the Millennium Development Goals by reducing the number of malaria cases by 75% (from ~ 600 to 150 thousand new cases) [25]. In the Brazilian Amazon from 2009 to 2020, a significant decrease in the new malaria cases has occurred in the states of Acre (− 55%), Amapá (− 74%), Amazonas (− 42%), Maranhão (− 98%), Pará (− 76%), Rondônia (− 70%), and Tocantins (− 100%); however, increasing trends are arising in Mato Grosso (+ 10%) and Roraima (+ 111%) states (Figs. 1, 2). Six clusters of epidemic municipalities befall Acre, Amazonas, and Pará states: cluster 1, Cruzeiro do Sul, Guajará, Ipixuna, Mâncio Lima, and Rodrigues Alves; cluster 2, Lábrea; cluster 3, Coari; cluster 4, Manaus and Rio Preto da Eva; cluster 5, Bagre and Oeiras do Pará, and cluster 6, Barcelos and Santa Isabel do Rio Negro (Figs. 3, 4, Table 1). Forecast analysis with these municipalities shows increased numbers in 2021 in Coari, Guajará, and Ipixuna (Fig. 5, Table 2). Imported malaria amount to 2.5% of all new malaria cases diagnosed and Venezuela is contributing with the recent cross-border malaria burden (Fig. 6). Lastly, new malaria cases observed in 2020 and the predicted numbers in 2025 and 2030 are all far from the targets proposed by the WHO GTS milestones (Fig. 7).

### Stable, resilient, or transient epidemic municipalities

In this study 40 municipalities were classified either as stable (6% or 15%), resilient (7% or 18%), or transient (27% or 67%) hotspots. Stability was observed when no malaria interventions were able to disturb the stable endemic cycles of malaria transmission in Cruzeiro do Sul-AC, Guajará-AM, Lábrea-AM, Manaus-AM, Mâncio Lima-AC, and Rodrigues Alves-AC. Resilience to malaria interventions was seen in Bagre-PA, Barcelos-AM, Coari-AM, Ipixuna-AM, Oeiras do Pará-PA, Rio Preto da Eva-AM, and Santa Isabel do Rio Negro-AM. In these municipalities, malaria occurrence was at nonequilibrium in-between two phases: (1) stabilizing phase, when the number of new malaria cases reaches an endemic level, and (2) destabilizing phase, when malaria interventions are able to curb the number of new cases to zero or near zero. Transience in the remaining 27 municipalities was composed of oscillatory increase and decrease towards equilibrium (no malaria cases).

As pointed out by Lana et al. [21], stable malaria transmission in Brazil occurs in the top 1% of Amazonian municipalities. Here, the 13 abovementioned municipalities represent 1.6% out of 808 Amazonian municipalities, altogether they reported 31% (731,179) of all autochthonous malaria cases (2,363,779) in the Brazilian Amazon in 2009–2020. These municipalities are the current challenge to malaria elimination in Brazil. Some countries have managed to move towards elimination with existing tools and interventions, such as Suriname in South America [26]. To reach the elimination goal, it is imperative to understand where malaria transmission occurs; for instance, in Brazil, malaria occurs in a scenario of high spatiotemporal heterogeneity, as shown in Table 1 and Fig. 3. Consequently, it will be necessary to have a strong political commitment and financial investment to eliminate the myriad of transmission foci [27, 28].

Looking at the malaria transmission cluster variation in space and time (Fig. 3, Table 1), the main interpretation is that the most endured hotspot in Brazil is in the Juruá River Valley, composed by Cruzeiro do Sul, Guajará, Mâncio Lima, and Rodrigues Alves, adjacent to neighbouring municipalities (Ipixuna). The basic reproduction number of *P. vivax* in agricultural settlements in Cruzeiro do Sul can be as high as 10 (one person infected by *P. vivax* can cause up to 10 new infections) [29]. The genomic signature of *P. vivax* populations in Mâncio Lima is characterized by high levels of inbreeding at local distances [30], which means the influence of people mobility is not so high on malaria transmission [31], and thus local of residence is still the major determinant for contracting malaria [32]. Living in precarious housing in the peripheries of the town’s urban centre is consistent with substantial transmission foci [33]. Individuals living in these high-risk settings may develop clinical immunity over time and become asymptomatic parasite carriers that can silently propagate malaria transmission [34]. These individuals should therefore be a priority target for interventions to decrease the endurance of malaria transmission in the Juruá River Valley and beyond.

A second interpretation that arises from the results of time-series analysis (Figs. 4, 5, Table 2) is that malaria transmission may reappear after the implementation of interventions such as mass drug administration and/or distribution of long-lasting insecticide-treated bed nets [35, 36]. The re-emergence represents a challenge for maintaining the success of malaria elimination. A recent study revealed the occurrence of a second malaria peak event after 35 years of colonization in rural settlements in the Brazilian Amazon [37]. Asymptomatic human carriers tied to the dominant malaria vector behaviour of both indoor and outdoor biting are underlying mechanisms of malaria re-emergence [38, 39]. In a modelling study, the risk of nonimmune travellers acquiring malaria is 13% in Mâncio Lima, 6% in Rodrigues Alves, and 4% in Cruzeiro do Sul, based on an 1-month visit [39].

Asymptomatic human carriers of *P. vivax* are overlooked by routine surveillance, but they can contribute to malaria transmission. The testing of new drugs to tackle malaria subpatent infection in asymptomatic human carriers remains the key to the malaria elimination target in Brazil [40–43]. Chloroquine is the first line treatment to *P. vivax* malaria in Brazil; however, two major drawbacks are associated with it: (1) *P. vivax* resistance to chloroquine is emerging in malaria transmission hotspots [44] and (2) *P. vivax* liver-stage hypnozoites cause relapsing infections that are only cleared with primaquine whose poor treatment adherence undermines radical cure effectiveness [45]. The alternative drug tafenoquine has shown potential to improve effective radical cure through increased adherence and thus to reduce new infections [45].

### Complexity and diversity of cross-border malaria

An important side-effect of asymptomatic infectious carriers is the exportation of malaria from another country to the Amazon basin, or vice-versa. This event has been defined as cross-border malaria and can have significant impact on the surveillance systems. Figure 6B shows that 51 municipalities diagnosed and treated free-of-charge 59 480 imported malaria cases from 2009 to 2020 in the Brazilian Amazon. While the ratio *P. vivax*/*P. falciparum* is 8/1 in autochthonous new malaria cases, it is 3 *P. vivax*/1 *P. falciparum* in imported malaria scenario (Fig. 6A). This indicates further complexity of eliminating *P. falciparum* malaria in Brazil. Despite the first line treatment with artemisinin combination therapies against *P. falciparum* malaria is effective, Fig. 6A shows that *P. falciparum* is constantly being imported to Brazil from neighbouring countries. Figure 6C shows that French Guiana was the source of high numbers of imported cases from 2009 to 2014 and Fig. 6D shows that Venezuela was the source in 2015–2020.

Cross-border malaria between French Guiana and Brazil is characterized by the continuous movement of people who undertake illegal mining activities in the artisanal gold mines of French Guiana and in the indigenous areas of Oiapoque in the Amapá state [46, 47]. Figure 6B shows that 11.3% (6704) of all imported malaria cases (59,480) in the Brazilian Amazon 2009–2020 occurred in Oiapoque near the French Guiana border where numerous spots of illegal mining have been georeferenced. One of the main issues with the cross-border malaria is the fragility of local health services in timely identifying and treating *P. vivax* and *P. falciparum* asymptomatic human carriers [47]. A recent development of a monitoring system may overcome the cross-border obstacle for malaria elimination [48]. This monitoring system tool is a visualization dashboard of time series and maps of epidemiological indicators based on data that is updated monthly. This tool is available to all parties involved in malaria control in Brazil and French Guiana [48]. Additionally, a study sought to understand gold miners’ perceptions in the municipality of Calçoene, near Oiapoque, found that targeted educational material can help them in the protection against malaria [49].

The high number of malaria cases imported from Venezuela in 2015 to 2020 shown in Fig. 6D is a warning to neighbouring countries in South America. The recent Venezuelan humanitarian crisis caused an unprecedented exodus to neighbouring countries [8], and this is an issue that will likely occur for several years in the future [10, 50–52]. The contribution of intensive refugee fluxes and mining activities along the Venezuela-Guyana-Brazil border clearly reinforces the need for malaria surveillance policies with robust strategies to detect the routes of movement of these mobile and vulnerable populations [53]. Cross-border infections can disproportionately affect hard-to-reach rural communities with poor access to health services [54–58]. Movement of asymptomatic human carriers should be tracked by surveillance [59]. This policy would help detect *Plasmodium* infections with the objective of treating the cases in a timely manner to interrupt the transmission chain and, consequently, decrease the malaria burden in the state of Roraima in Brazil. Table 1 shows four municipalities in this state (Alto Alegre, Amajari, Iracema, and Mucajaí) that have become new clusters of malaria transmission.

### Pitfalls in disease elimination: the stretch goal paradox

Considering the results in Fig. 1A, the number of new malaria cases dropped from 301,581 in 2009 to 142,107 in 2020 (− 53%). The numbers of new *P. vivax* and *P. falciparum* malaria cases in the same period were also reduced, respectively, by 53% (118,959/253,655) and 52% (23,033/47,841) (Fig. 1B). This reduction is, however, not sufficient to comply with the WHO GTS targets (Fig. 7). This outcome rather frustrating is caused by a combination of pitfalls in disease elimination that can be illustrated by the stretch goal paradox [60]. Stretch goals are viewed as truly important sources of goal achievement, but this is far from the truth as they are often extremely difficult to achieve or they depend on brand-new technologies that are not readily available [60]. Setting an extremely difficult goal rather than an achievable objective can trigger negative attitudes and actions. Figure 1A shows that the number of new malaria cases in Brazil changed its direction and started increasing from 2017 on, just after the WHO had proposed the GTS for Malaria 2016–2030. Alternatively, this reversing trend in Fig. 1A may be related to additional challenges coming to light and shifting attention from malaria elimination goals. This priority shift can jeopardize the organization of health services that carry out the diagnosis and treatment of malaria cases.

The health surveillance programme in Brazil is internationally recognized for having controlled malaria and attained a decline in malaria mortality rates [12, 13, 53, 61, 62]. The 2003 National Malaria Prevention and Control Program (NMPCP) launched by the Brazilian Ministry of Health helped significantly decrease malaria deaths and severe cases in the 2000s [13]. The fight against malaria can be seriously prolonged or slowed when there is a lack of robust, predictable, and sustained financial commitment [1]. For instance, financial issues occurred during the Global Malaria Eradication Program (GMEP) in the 1960s–1970s [63]. However, when a strong political commitment is compounded by a consensual priority of eliminating malaria, a country can succeed in controlling the disease. This scenario occurred at the beginning of the NMPCP implementation in the 2000s when the Brazilian federal administration, states, and municipalities delineated the national agenda for malaria control by structuring and organizing local health surveillance services [64]. Political instabilities, as seen now in Venezuela and Brazil, lead to the failure of control programmes, resulting in an intensification of malaria transmission [46, 47, 62].

## Limitations

The major limitation with analyses carried out using municipality-based datasets is the spatial scale of malaria transmission. Amazonian municipalities are often very large and are sparsely populated, with high spatial clustering of people where malaria transmission often occurs in spatial scales of 5-km^2^ [65]. Here we sought to overcome this limitation by analysing the total number of new malaria cases per country, state, and municipality. The total number of new malaria cases is assumed as a proxy to the magnitude of the spatially clustered foci of malaria transmission in each administrative boundary in the period analysed.

## Conclusions

Unbeatable malaria transmission occurs in six municipalities (< 1%) in the Amazon region. Four of them (67%) are in the Juruá River Valley (Cruzeiro do Sul, Guajará, Mâncio Lima, and Rodrigues Alves). Importation of *P. vivax* and *P. falciparum* from neighbouring countries (particularly French Guiana and Venezuela) further complicates elimination. The lack of achievement of the 2020 WHO GTS target is either related to the stretch goal paradox or shifting priorities from the federal government. In any case, the achievement of the malaria elimination goal by 2030 is unlikely. An enhanced political commitment is key for the post-2030 malaria elimination milestones in Brazil.

## Supplementary Information

**Additional file 1.** New malaria cases per municipality of infection, Brazilian Amazon, 2009-2020.

**Additional file 2.** Monthly new malaria cases per epidemic municipalities, Brazilian Amazon, 2009-2020.

**Additional file 3.** Imported malaria cases per bordering country of infection, Brazilian Amazon, 2009-2020..

**Additional file 4.** Imported malaria cases per municipality of official case report, Brazilian Amazon, 2009-2020.

**Additional file 5.** Imported P. falciparum-malaria cases per municipality of official case report, Brazilian Amazon, 2009-2020.

**Additional file 6.** Total new malaria cases in the Brazilian Amazon per month from January/2009 to December/2020.

**Additional file 7.** Total new malaria cases in the Brazilian Amazon per parasite species, 2009-2020.

## Data Availability

The datasets analysed in the current study are available in the Additional files 1, 2, 3, 4, 5, 6, and 7.
